# Supplementary material for: Are carbon emissions trading and green financial instruments synergistic? -Comprehensive quantitative research based on content analysis
Source: PLoS One. 2024 Mar 7;19(3):e0298601. doi: 10.1371/journal.pone.0298601 (PMC10919601; doi:10.1371/journal.pone.0298601)
Supplement: S1 Data — (ZIP) [file pone.0298601.s001.zip › Data/地级市Z_F工作报告.zip!/░▓╗╒/│n╓▌╩╨/└┤╫╘╣1⁄2╓┌║┼í╛┬φ┐╦╩2╛▌═°í┐_2011.docx]

各位代表：

　　现在，我代表市人民政府，向大会作政府工作报告，请予审议，并请市政协委员和其他列席人员提出意见。

　　一、2010年和“十一五”经济社会发展情况回顾

　　2010年是“十一五”的收官之年，也是大滁城建设突破之年、工业化进程加快之年、示范区机遇抢抓之年。一年来，全市上下在省委、省政府和市委的坚强领导下，深入贯彻落实科学发展观，奋力拼搏，扎实工作，实现了经济实力的新跨越，社会事业的新发展，和谐社会的新局面。

　　一是六大总量指标迈上新台阶。生产总值跨过600亿台阶，预计690亿元以上，年增量首超百亿；财政收入连跨三个十亿元台阶，达到90.5亿元，在全省位次前移一位，年增量在连续三年保持10亿元左右的基础上，首超20亿，达到22亿元，市本级全部消化历史遗留的赤字包袱，甩掉戴了6年的“财政赤字市”帽子；工业总产值跃上千亿大台阶，达到1059亿元，当年新增373亿元；全部工业增加值迈上300亿台阶，其中规模以上工业增加值296亿元；社会消费品零售总额迈上200亿台阶，达到215亿元；进出口总额年增量超过2亿美元，总量连跨三个亿美元台阶，达到9.1亿美元，稳居全省进出口额前五名、出口额前三名。

　　二是三项增幅指标达到新水平。预计生产总值比上年增15 %以上，财政收入增32.4%，规模以上工业增加值增27%，分别超年初计划1.5个、14.4个和6个百分点，均创十多年来最好水平。

　　三是三套结构指标得到新优化。三次产业中工业主导地位更加凸显。工业增加值占生产总值的比重首次突破40%，达到44%。财政收入占生产总值比重超过13%，加速了农业财政向工业财政的转变。板块结构加快优化，县级财政收入占全市财政收入比重上升到62.6%，县域经济对全市经济增长的贡献率进一步提高。

　　四是两类生活指标实现新提高。全市城镇居民人均可支配收入达15100元，农民人均纯收入达5600元，分别增长13%和11.5%，生活水平进一步提升。

　　（一）千方百计扩大投资，拉动发展增后劲

　　在统筹发挥投资、消费、出口“三驾马车”拉动作用的同时，把强化投入作为主动力，争投资解决“有钱可投”，抓项目促进“有钱快投”，扩园区保证“有地可投”。

　　多管齐下争引投资。一是抢抓产业转移机遇，强力招大引强。共引进项目2350个，总投资1870亿元。其中到位资金624亿元，同比增加200亿元，增长45.8%；新引进项目中亿元以上216个，5亿元以上50个，10亿元以上24个，50亿元以上1个。六大工业支柱产业到位资金400亿元，占64%。深化与央企合作，新引进项目20个，总投资171亿元；开展与知名民企对接，签订合作项目188个，总投资超过700亿元，居全省第三位。二是抢抓扩大内需的机遇，积极向上争取项目。共争取上级项目资金60亿元，其中农业和农村经济项目28.4亿元，工业和科技项目4.4亿元，服务业项目2.7亿元，社会事业项目11.4亿元。三是加强银政企合作。年末贷款余额475亿元，新增贷款再超百亿，达103亿元，增速继续位居全省前列。四是发挥政府投资的引导性作用。全年完成政府性投资超过200亿元，同比增长近一倍。五是动员全民创业，鼓励民间投资。新增私营企业3219家、个体工商户4363户、注册资本26.4亿元。

　　扩增园区承接投资。开展市开发区二次创业，财政收入增长44%，规模以上工业增加值增长50%，固定资产投资和招商引资到位资金分别增长60%，创省级高新区进展顺利，创国家级开发区已上报国务院待批。扩增扩容省级园区。琅琊经济开发区、定远盐化产业园获准筹建，全市省级工业园区发展到13个，总体规划面积从178平方公里扩展到500平方公里以上。加快园区基础建设，新开发面积38平方公里。滁州承接产业转移示范园区完成150平方公里概念性规划、42平方公里核心区总体规划、20平方公里起步区控制性详细规划，起步区建设与招商同步展开。探索园区合作共建。达成20多个共建协议，宁滁高新园等13个合作园已开工建设。建成农民工创业园24个。

　　抓住项目实现投资。实施重大项目攻坚年活动，排出60项重点工程，领导牵头，部门承办，效能督查，强力推进。开展“月月都是开工月、投产月”和“三看三比”活动，推动重大项目快建设、快投产。全市1000万元以上项目新开工1122个，新竣工投产525个，其中亿元以上77个；完成“861”项目投资290亿元，超额完成计划的80%，居全省第四位；央企合作项目13个开工建设，完成投资70亿元。固定资产完成投资703亿元，增长38.8%，总量和增速分居全省第6位和第4位，拉动经济增长8.7个百分点，贡献率达58%。滁河综合治理获国家批准，大唐滁州发电厂等重大项目取得积极进展。

　　（二）培育壮大三次产业，扩张总量增实力

　　突出工业主导、农业基础、服务业带动，加快构建现代产业体系，优化产业结构，推动三次产业快速发展和提升。

　　突出发展工业经济。扩存量、提增量并举，大力实施小企业上规模、中企业上水平、大企业上台阶工程。规上企业发展到1299户，新增292户，年新增量是常年的两倍以上，其中约有150户来自对原有小企业的培育和扶持。中企业上水平，坚持政府引导和企业技改相结合，领导联系、重点帮扶、基础配套、政府贴息并举，力促提升产能和档次，完成技改投资150亿元，增长28.2%，政府贴息5000万元。天大管材二期、南谯恒昌机械、琅琊霞客色纺、明光酒业等62个5000万元以上重大技改项目竣工投产。全市年产值亿元以上企业发展到209户，当年新增69户。重点扶持大企业上台阶，锻造产业脊梁，紧紧围绕大企业及其产业链抓招商，力促产业集结。

　　重点规划和培育了家电信息、硅玻璃、盐化工三大千亿产业，农副产品精深加工、装备制造、新能源等超百亿产业，以及博西华、天大、全柴、天康、安康等一批目标百亿的企业。三大千亿产业中，家电信息产业引进了博西华新基地、康佳白电、格兰仕、东菱凯琴等品牌项目，实现产值超200亿元，获国家级“家电及装备制造业基地”和“家电设计与制造特色产业基地”称号。硅玻璃产业完成产业园基础设施投资4.1亿元，建成省级玻璃制品检测中心，总投资30亿元的中建材凤阳硅材料产业园开工建设。盐化工产业，一期投资75亿元的定远华塑盐化项目累计完成投资40亿元，泉盛化工百万吨纯碱等项目加快推进。全椒熔盛重工、天长吉阳科技、来安龙源风电、市开发区长电科技等一批投资20亿元以上大项目纷纷入驻。去年年产值超10亿元企业达14户，比上年增加5户。

　　大力发展三产服务。开展家电下乡、“以旧换新”和汽车、建材下乡四大活动，促进城乡消费。家电下乡销售超过50万台，销售收入超过11亿元，均是上年4倍以上。突出抓好商业综合体和大型专业市场。中环国际广场、长江财富广场、城市星座、泰鑫现代城4大城市商业综合体开工建设，5个农贸市场提标升级。大雄农贸食品城、瑶海农机大市场、滁州国际商城、奥特莱斯直销广场加快建设，来安金太阳建材市场、全椒盛大装饰城、天长天润城家居广场建成使用。加强旅游景区和酒店建设。九天峰、龙通度假村等续建项目进展良好，神山、大柳度假村前期工作顺利推进，栖凤湖、跃龙湖、龙尾山度假中心和丰乐亭景区综合整治项目开工，红花湖、金牛湖度假中心正式签约。全市已建成四星级以上标准的酒店8家，其中五星级1家；在建和拟建四星级以上标准的酒店22家，其中五星级9家。全年旅游接待640万人次，旅游业总收入突破40亿元，同比分别增长26.3%和25.6%。着力抓好金融机构引建和企业上市工作。新引进交通银行和江阴农商行，徽商银行凤凰支行、中行凤阳支行开业，全椒和明光农村信用社改组为农村合作银行，市郊联社获批筹建皖东农村商业银行，定远、南谯、全椒、来安和天长村镇银行加快组建，全市小额贷款公司新开业8家，新获批筹建3家。企业上市工作取得重要进展，德力玻璃、金禾化工上市上报国家证券会待批，扬子木业完成股份制改造。保险业继续发展。加快发展房地产业。预计全市房地产施工面积1142万平方米，其中新开工面积628万平方米，销售面积283万平方米，完成投资131亿元，分别增长81.5%、96%、33%和98.5%，均居全省前列。

　　积极发展现代农业。百亿粮仓再传捷报。粮食产量86.2亿斤，连续五年创新高。深入开展小麦高产攻关、水稻产业提升等行动，推广超级杂交稻188万亩。完成1个大型水库、25座中型水库和75座小型水库除险加固。规模养殖成为养殖业发展的主导力量。全市专业养殖户超过万户，其中千头养猪场270个、万只养禽场424个，规模养殖比重超过70%。肉类总产37.4万吨，增长3.4%。水产品产量27.4万吨，增长5.6%。发展特色农业，种植蔬菜77万亩、甜叶菊10万亩、滁菊8000亩，建成了一批生态农业、观光农业基地。发展农业装备体系，农机总动力达到576万千瓦，居全省第四位。加强农技推广体系，建成测土配方施肥数字库，建设8个现代农业示范区，良种良法覆盖率超过80%。延伸农业产业化体系，市级以上龙头企业达到121家，其中省级以上43家，新增无公害农产品、绿色食品和有机食品共129个，总数达到367个。培育新型农业生产体系，规模种植大户发展到近3000户，农民专业合作社发展到1355个，集体林权制度改革确权发证全面完成，农村土地流转面积新增11万亩，总量达74万亩。

　　（三）强力推进城镇建设，统筹城乡求突破

　　以大滁城建设为龙头，丰乐杯城镇竞赛、农村土地整治复垦为抓手，大力开展城乡建设，完成政府性投资200多亿元（主城区103亿元），是前两年总和的两倍多，其中规划编制投入超亿元。

　　大滁城建设初见成效，展现新貌。目标定位再提升。明确了建设合宁之间新型区域中心城市的目标。功能布局再优化。主城区调整扩大了市经济技术开发区，谋划设立了示范园区、高铁站区、科教园区、琅琊新区、琅琊开发区，并完成了各区规划。组团建设再突破。主城区实施政府投资项目146个，新建续建道路40条103公里，框架继续拉开；政务中心基本建成，新滁州中学等三所学校建成使用，龙蟠小区、天逸华府、发能国际城、同乐苑等小区数十幢高层建筑拔地而起，城南新区初见雏形；高铁站区路网、站前广场、公交枢纽开工建设，对外通道进一步打开；科教园区内农机校、交通技校开工建设；老城改造密集展开，完成拆迁173万平方米，开工建设安置房233万平方米，明光路跨线桥加快建设。通过努力，主城区路网拓展到70平方公里以上，城市变得更大了。主城区已建、在建和已批准待建高层总数达629幢，其中18层以上238幢，城市变得更高了。深入开展“五城联创”，实施“五小工程”；菱溪湖公园、龙蟠河公园、南湖公园二期、同乐坊全面建成；清流污水处理厂二期提前竣工；新增绿地218万平方米，城市绿化覆盖率达到39%，省园林城市创建通过专家考评；完成丰乐大道和清流西路示范改造，高标准实施清流西路、南湖等亮化工程，城市变得更美了。与此同时，四大组团齐头并进。琅琊新区基础设施建设全面启动，南谯新城完成投资30亿元，全椒城北新城完成规划，来安新区加速与滁城对接，滁来路完成高标准改造。作为滁州人民政治经济文化中心的大滁城正在加快展示新形象。

　　各县城建设你追我赶，广获好评。各地围绕新城区拉框架增容量，老城区抓改造变环境，大力开展县城建设，年度投资首超百亿。天长市75个城建项目完成投资20亿元，投资近亿元的荭草湖公园基本建成；定远县30个城建项目完成投资30亿元；明光市33个城建项目完成投资12亿元；全椒县43个城建项目完成投资26亿元，投资6亿元的襄河治理及风光带项目开工建设；来安县50个城建项目完成投资12亿元；凤阳新城完成投资12亿元。

　　新农村建设纵深推进，翻天覆地。以农村土地整理和建设用地置换为契机，大力推进新农村建设。2009年以来累计申报建设用地置换面积11.8万亩，占全省三分之一，获批9.5万亩，通过验收7.3万亩。省批准的17万亩土地治理项目基本完成。新申报的整体推进农村土地整治30个示范项目共46.6万亩，已有4个开工。通过土地整治复垦，新增耕地近4万亩。大整治开拓了新农村建设的新空间。全市新农村居民点已达727个，其中去年新建382个，已入住1.34万户。

　　大交通建设加快发力，区位更显。交通建设完成投资13.6亿元。徐（州）明（光）高速公路滁州段完成征地拆迁，宁洛高速公路来安至明光间的延伸段开工建设，滁（州）马（鞍山）高速公路前期工作积极推进。完成合蚌路、滁定路改善续建，307省道、104国道大修竣工。建设村级道路545公里。京沪高铁滁州段完成铺轨，定远青岗站获批，成为全线唯一拥有两个高铁站的地区。滁宁轻轨被列入长三角城际高速铁路规划。此外，全市电力建设完成投资10.4亿元、通信建设完成投资3亿元。

　　（四）大力发展社会事业，保障民生促和谐

　　社会保障齐头并进。全市新增就业人口5.4万人，增长80%，超计划64%。社会保障提标扩面。职工基本养老保险人数增加到33万人、基本医疗保险增加到33.8万人、失业保险增加到21万人、生育保险增加到17.6万人、工伤保险增加到20.7万人。失业保险和工伤保险实现了市级统筹。城镇居民基本医疗保险和新型农村合作医疗保险基本全覆盖。城乡居民最低生活保障实现了动态管理下的应保尽保和分类施保。提高了城乡低保标准和最低工资标准。调整出台了失地农民保障政策，7.9万被征地农民纳入养老保障范围。按照国家和省规定，向优抚对象、城乡低保家庭、在校大学生共发放1785万元临时价格补贴，对事业单位职工预发了生活补贴，提高了公务员津贴标准。

　　社会事业加快发展。全市财政教育投入20.2亿元，教育经费得到有效保障。全面启动中小学校舍安全工程，投入6.15亿元，完成重建和加固102万平方米。滁州中学、滁州六中、实验小学、全椒中学迁入新校区，滁州职业技术学院二期、天长炳辉中学初中部建成投入使用。省级技术中心达到32家，国家级1家。完成高新技术产值420亿元，获批专利1037件，分居全省前五位和前三位。新组建滁州广播电视台和市演艺公司，在全省率先突破文化体制改革，受到省委、省政府表彰。新建乡镇文化站27个、农家书屋154个。精心承办第三届中国农民歌会，获得部省主要领导高度评价和全国社会文化艺术政府奖第十五届群星奖。基层医改全面推开。实行了药品销售零差率，群众就医满意度提高，实现了转制无震荡、零上访。新建和改造乡镇卫生院5所、村卫生室518个、社区卫生服务站13个、妇幼机构2个，市一院和中西医结合医院医技大楼基本建成。公益性卫生体系加快形成。

　　社会管理得到加强。人口计生提升工程高强度推进，“村为主”经验得到国家计生委肯定。全面开展第六次人口普查。防震减灾工作获得国家一等奖。强化安全隐患排查整治，安全生产形势进一步好转。滁城尾砂坝综合治理工程竣工，城西水库除险加固通过验收，原子能研究所钴60放射源安全迁出，困扰滁城居民多年的“四大安全威胁”全部排除。加强应急管理，公共突发事件得到有效防范。加强平安滁州建设，严厉打击各类违法犯罪，强化社会治安综合治理。推进县市区联合接访大厅、乡镇维稳综治办和村（社区）维稳综治站建设，完善群众工作和矛盾纠纷化解长效机制。深入开展“三民”工程大走访活动，努力化解社会矛盾。法律援助工作全省综合考评保持第一。“五五”普法顺利通过省级验收。国防动员工作荣获南京军区先进。“阳光村务”工程大力推进并在全省推广。48个村被授予省民主法治先进村称号。人民防空建设力度加大。争创全国“双拥”模范城四连冠扎实开展。安定团结、和谐稳定的社会局面得到巩固。此外，民族宗教和侨务、统计、档案、科普、妇联及工商联等工作都有新的进展。

　　民生工程扎实推进。36项民生工程投入资金23.5亿元，全面完成工作任务。解决了60万农村人口饮用水安全问题。新建成农民健身工程点67个、广播电视“村村通”工程593个、农村留守儿童之家383个、农村五保供养机构31个、儿童福利院和光荣院各1个。为全市42.7万名农村中小学生和城市低收入家庭学生提供了免费教科书，为7509名中小学寄宿生提供生活补助544万元，资助中职学校家庭困难学生4.2万人次3185万元。免费为贫困白内障患者做复明手术854例，向贫困重度残疾人发放生活特别救助2443万元。培训各类农民6.1万人。全市投资96亿元开工建设安置房419万平方米，已建成1.7万套、169万平方米，5500多户居民迁入新居；保障性住房惠及居民11659户，占省下达任务的137%；改造棚户区住房11267套，占省下达任务107%。

　　节能环保得到强化。适应国家节能节电双考核要求，加强调度督查，全面完成44家重点企业节能任务，淘汰52条落后水泥生产线，推广高效照明产品26万只，综合能耗下降4.5%。以创建国家环保模范城市为契机，加强污水处理厂和管网建设，完成投资1.9亿元，新建污水管网220公里；开展国省控重点企业和减排项目专项整治，化学需氧量减排4200吨，二氧化硫减排2300吨。推进生态市建设。完成植树造林15.8万亩。清理闲置土地，整治违规用地。全面完成基本农田保护任务，受到国土资源部表彰。进一步整合矿产资源，矿山开采企业减少37家。

　　（五）坚持从严治政，提高效能优环境

　　坚持依法行政。主动接受市人大及其常委会的法律监督和工作监督，自觉接受市政协的民主监督。市人大议案3件、代表建议44件和政协委员提案362件全部按时办结。着眼于规范行政权力运行，制定了市政府八项工作制度，废止规范性文件143件。市县两级政府机构改革全面完成。加强机关效能建设。深化“两集中”、“两到位”改革。完善基建联合审批、企业登记注册一表制、“3+1”联网审批、政府信息公开四大平台。市本级新组建招商信息处理、招商项目谈判、项目建设帮办、投产企业帮扶四大服务中心，安排200多名干部帮办98个项目、帮扶115个企业。建立滁州民声网，办好市长信箱和“12345”热线，开展网络问政。开展社会评窗口、群众评项目、企业评部门、万人评科长“四项评议”活动，开设《效能观察》、《效能跟踪》栏目，强化社会监督。认真落实政府系统党风廉政建设责任制，加强反腐败惩防体系建设，政府廉政建设不断深入。

　　2010年的发展，为“十一五”画上了圆满的句号。回首“十一五”，发展突飞猛进，成就令人鼓舞。

　　一是综合实力实现了成倍扩张。地区生产总值和人均地区生产总值在“十五”期末基础上实现翻番，人均地区生产总值超过1.6万元。财政总收入是“十一五”规划60亿元目标的1.5倍，总收入和地方财政收入分别比“十五”末增长2.3倍和3.8倍。

　　二是产业结构得到了明显优化。三次产业结构比例调整到21.2:47.6:31.2，与“十五”末相比，一产下降6.3个百分点，二产上升9.6个百分点。农业产业化步伐不断加快。工业化率比“十五”末提高12个百分点，工业对经济增长的贡献率达到66.5%。服务业增加值比“十五”末增长了1.8倍。

　　三是基础设施获得了快速提升。以防洪、灌溉、供水、生态保护为主的水利工程体系初步形成。综合交通网络初具规模，公路通车里程比“十五”末增长1.5倍。通信网络覆盖城乡，“数字滁州”框架建立，能源保障得到强化，电网规模进入全省第一方阵。

　　四是城乡面貌发生了显著变化。全市城镇化水平提升到42.8%，年均提高近2个百分点。“大滁城”“131”组团框架不断拉大，功能不断增强，布局渐趋合理，形象不断改善；全椒、来安县城与主城区同城化进程加快。全市城市建成区面积达到200平方公里，比“十五”末扩大77平方公里。县域板块建设迈出坚实步伐，县城面貌普遍改观，经济强镇脱颖而出，一大批新农村居民点快速涌现，农村新社区呈现新面貌。

　　五是对外开放呈现了崭新格局。“十一五”期间，全市招商引资到位资金累计突破1600亿元，年均增速超过50%；实际利用外资累计达到7.3亿美元；对外贸易额实现翻番。全市省级开发园区增加7个，建成区面积扩大近6倍，新发展的35个乡镇工业集中区建成面积83平方公里，成为推进开放、统筹城乡、以工补农、以城带乡的前沿阵地。

　　六是改革创新增添了强大动力。创新体制机制，建设服务型政府，软环境显著改善。农村综合改革进一步深化。扩权强镇试点扎实开展。五年完成近千户国有集体企业改制，特别是去年强力推进企业改革攻坚年活动，基本完成105户企业改革，确保了省“十一五”企业改革规划中我市任务基本完成。非公有制经济比重逐年提高。地方金融体系进一步健全。内生动力明显增强。

　　七是“生态滁州”完成了繁重任务。荣获“2010中国城市科学发展转变经济发展方式示范城市”称号。森林覆盖率上升到25.2%，五年增加4.8个百分点。切实抓好重点耗能行业企业的节能降耗。市及每个县市建成了污水处理厂。国控、省控、市控污染源实现了在线监控。城镇饮水达标率100%，农村自来水到户率上升到42%。节能减排目标全面完成。

　　八是民生福祉取得了明显改善。以提高人民生活水平为第一目标，大力实施民生工程，累计投入66.8亿元，惠及400多万城乡居民。实施积极就业政策，五年城镇新增就业17万人。社会保障制度体系初步形成。教育资源质量和布局明显改善。文化广电新闻出版事业蓬勃发展。公共卫生服务水平得到提高。城镇居民人均可支配收入和农村居民人均纯收入年均分别增长12.6%和14.9%，居民储蓄存款年均增长16.5%。精神文明和民主法制建设不断加强，社会保持和谐稳定。

　　各位代表，“十一五”是滁州发展极不平凡的五年，我们战胜了冰雪、洪涝等多种灾害和国际金融危机的强烈冲击，取得了超出预期的发展成就。成绩来之不易，这是科学发展观在滁州大地生动实践的结果，是省委省政府和市委坚强领导的结果，是全市上下奋力拼搏的结果！在此，我代表市人民政府，向全市人民，向人大代表、政协委员和党代会代表，向市县两级几大班子历任领导和离退休老同志，向各民主党派、工商联、无党派人士和各人民团体，向驻滁解放军指战员、武警消防官兵和政法干警，向国家和省驻滁单位，向广大来滁投资者和建设者，向所有关心支持滁州发展的海内外朋友，表示衷心的感谢和崇高的敬意！

　　在肯定成绩同时，我们也要认清形势、看到困难、直面问题。

　　从内部看，我们还存在很多问题和不足。一是经济上，总量不够大、结构不够优、层次不够高，企业不够强、项目不够好，一产不够壮，三产不够快。二是城建上，主城区首位度不够高、功能不够全、带动力不够大，纵向比变化巨大，横向比差距明显；县城面貌老化，建设步伐滞后，发展功能缺失，产业发育不够全，居住环境不够优。三是民生上，财政保障能力比较弱，社会事业“欠账”比较多，群众反映的企业改革、征地拆迁、环境保护等方面的问题还不少。四是在干部上，政府系统自身建设还存在薄弱环节，在招商引资、城镇建设等方面解放思想的力度还不够大、程度还不够深，政府机关在服务群众、服务企业上还存在效率不高、方法简单、态度傲慢甚至吃拿卡要现象，公务员队伍的市场经济意识、工作创新能力、风险控制水平、应对复杂局面经验等都还存在不足，腐败个案时有发生，廉政建设任重道远。

　　从外部看，我们还面临很多困难与挑战。一是来自被“边缘化”的挑战。我们周边大城市林立，同时处在合肥都市圈外、合芜蚌自主创新试验区边，虽地跨长江、淮河两大流域，但又不属于皖江城市带、沿淮城市群的主体城市，“配角”地位明显。如果长期处于落后，自然资源、消费市场、各类人才及劳动力等被周边发达地区吸附或“挤压”，必将导致产业低端，市场萧条，面临被“边缘化”的危险。二是来自“国际化”的挑战。经济全球化不可阻挡，特别是经过这一轮金融危机的打击，国际经济潜藏着大量不确定、不稳定、不明朗的因素，我们作为欠发达地区，内生动力不够强，在宏观经济波动时不可避免受影响。三是来自竞争“白热化”的挑战。当前区域竞争日趋“残酷”，招商引资、承接转移更是直接的政策环境大比拼，达到“白热化”程度。全市上下务必头脑清醒，咬紧牙关，直面挑战。

　　从条件看，我们还面临很多风险与制约。一是环境承载力制约。“十二五”期间节能减排仍以“十五”末为基期数，且要求更严格，我市“十五”发展基数低，能耗和排放量水平低，在低基数的基础上进一步降耗减排压力很大。同时，我们依托资源规划的盐化工、硅（玻璃）等产业相对耗能高，低基数与高耗能的较大反差，加之发展的巨大压力，处理不好就容易使我们陷入“头痛医头、脚痛医脚”的两难困局。二是土地供给制约。“十二五”期间，国家将强化土地利用规划和年度用地计划调控，指标从紧，程序从严，监控从密，成本也会增加，用地难度将进一步加大。三是人力资源制约。我市劳动力资源比较丰富，但大量劳动力外流，招工难、就业难同时并存，市内各开发区迫切需要政府、学校、企业共同解决人力资源量怎样保证、质怎么提高、“桥”怎能架通、学怎以致用等诸多问题。

　　以上困难问题我们将认真对待，采取措施逐步解决或缓解。

　　二、“十二五”经济社会发展的指导思想、总体目标和思路

　　“十二五”是滁州奋力崛起的关键阶段、转型发展的重要时期。按照党的十七届五中全会和省委、省政府及市委的部署，“十二五”期间全市经济社会发展的指导思想是：高举中国特色社会主义伟大旗帜，以邓小平理论和“三个代表”重要思想为指导，以科学发展为主题，以转变经济发展方式为主线，以追赶跨越、奋力“双超”为主调，以省内争先进位、省外对标苏北平均水平为主目标，以扩大开放、改革创新为主动力，以兴滁富民和改善民生为出发点和落脚点，抢抓皖江城市带承接产业转移示范区建设重大机遇，大力实施工业强市、城市带动、承接合作、转型升级、科教兴滁五大战略，着力打造承接产业转移的新型产业园、与南京同城化的城市新家园、吸引东部的魅力后花园，努力进入全省发展第一方阵，全力把滁州建设成为与南京都市圈、合肥经济圈紧密对接，引领皖东跨越发展的新型区域性中心城市。

　　第一，经济社会目标全面实现。到“十二五”末，实现地区生产总值、固定资产投资、财政收入、服务业增加值、城乡居民收入“五个翻番”；民主法治素质、文化繁荣水平、社会保障能力、生态文明程度、人民生活质量“五个显著提高”。地区生产总值、财政收入年均分别增长16%和22%以上；总量分别达到1600亿元和250亿元，人均生产总值超5000美元，追赶跨越取得突破性进展。

　　第二，新型工业基地基本建成。家电信息、硅玻璃、盐化工三大主导产业产值分别达到800亿元、500亿元、300亿元，为2020年全面实现超千亿目标，成为全国重要的家电信息产业基地、华东地区规模最大的硅玻璃产业群和规模效益领先的盐化工产业群打下坚实基础；同时，基本建成农副产品精深加工、装备制造、新能源三个五百亿元级支柱产业，建成一批百亿元级基础产业；形成博西华、天大、全柴、天康、安康等一批超百亿的企业集团。产业结构明显优化。三次产业结构调整到12:52:36，工业化率达到50%以上。产业布局更趋合理。基本形成滁来全产业核心、定凤明资源精加工、天长先进制造三大片区的空间开发格局。

　　第三，新型中心城市初具规模。城镇人口达到240万人左右，城镇化率超过50%，百平方公里、百万人口的中心城区和一主三副的大滁城组团基本形成，为2020年大滁城达到200平方公里、200万人口打下良好基础。天长、明光、定远、凤阳发展成为30万左右人口的中等城市。依托重点镇建成一批经济发展、功能完善、环境优美的精品小城市。

　　第四，园区经济成为主要支撑。基本建成2-3个千亿园区、2-3个500亿园区、省级开发区均超200亿，一批工业集中区过百亿。市经济技术开发区建成产业集聚、特色鲜明、生态环保的国家级开发区，争取进入全省第一方阵；示范园区形成产城一体、综合性、多元化的现代产业城；高铁站区成为集商贸、物流、文化等现代服务业集聚区；科教园区成为“大滁城”的文化教育中心和全省一流的科教园区、长三角知名的职教园区；省级开发区和工业集中区成为县域经济崛起的主要支撑。

　　第五，转型升级步伐明显加快。新材料、新能源、电子信息、高端装备制造等新兴产业取得突破。现代服务业取得重大进展。大物流、大市场强劲发展，成为全国重要的物流节点城市和物流基地；以琅琊山为龙头的大旅游格局形成，成为华东乃至全国重要的现代休闲度假基地和旅游目的地。创新型城市建设取得实质性进展，科技创新体系初步构建。

　　第六，城乡统筹格局基本形成。工业向园区、农民向城镇、土地向规模经营“三集中”步伐加快，农业产业化、农村社区化、农民市民化“三化”取得重大突破，“百亿粮仓”基本建成，优势农产品基本实现区域化布局、专业化生产、企业化经营，成为泛长三角地区重要的优质农产品生产加工供应基地；城乡公共基础设施和公共服务基本实现一体化；一大批居住集中、设施完善的新型农村社区相继建成，成为农民幸福生活的美好家园。

　　第七，人民幸福指数大幅提高。城镇居民人均可支配收入达到30000元，农民人均收入达到12000元。城乡生态更加优美，人居条件明显改善，民生保障更加健全，社会建设和谐稳定。

　　做好“十二五”工作，要重点把握以下四点：

　　一是坚持把科学发展作为主题。以人为本是发展的前提和归宿。滁州又好又快发展的条件和机遇已经具备，既要加快发展，做大总量，更要可持续发展，提高质量，实现包容性增长。

　　二是坚持把加快转变经济发展方式作为主线。发展不快、发展不足、发展不优仍然是现阶段滁州最大的现实、最主要的矛盾，加快转变经济发展方式是突破土地和环境容量制约的现实选择，是产业结构升级的必由之路。

　　三是坚持把追赶跨越、实现“双超”作为主调。与一些发展快的地区相比，我们无论是经济增长速度、运行质量，还是综合实力的差距仍然没有缩小。要想在“十二五”赶超周边地区，必须以更快的速度、更大的步伐追先进、进前列，实现“双超”，使综合竞争力有更大提升、人民生活有更大改善。

　　四是坚持把工业强市、城市带动、承接合作、转型升级、科教兴滁作为主战略。一方面，我们正处在工业化、城镇化、区域经济一体化的加速期，必须坚持工业化、城镇化双轮驱动，主动参与区域分工；另一方面，转变发展方式必须加快转型升级，强化科教支撑。新的五大战略深化和丰富了“十一五”三大战略思想，是新一轮发展的重要引领。

　　三、2011年主要工作安排

　　2011年是“十二五”的开局之年，要围绕“十二五”的总体思路，省内争先进位，省外对标苏北，以“661”行动计划（打造6大支柱产业、实施60项重点工程、竣工100个亿元以上项目）为主抓手，大力开展园区建设突破年、重大项目攻坚年、城乡统筹推进年活动，奋力开好局、起好步。经济社会发展的预期目标是：生产总值增长16%，财政收入增长20%以上，全社会固定资产投资增长25%以上，社会消费品零售总额增长19%，外贸进出口总额增长12%，城镇登记失业率控制在4%以内，人口自然增长率控制在7‰以内，单位生产总值能耗降低2%，主要污染物排放量减少1.5%。

　　（一）在园区建设上突破，推动承接平台大扩张

　　今年是承接产业转移全面推进的关键之年，打好园区建设战役，谋求新突破、实现新提升成为重中之重。全市所有省级工业园区都要实现财政收入增长40%以上，规模以上工业增加值增长50%以上，固定资产投资和招商引资到位资金分别增长60%以上。

　　加快园区扩张步伐。狠抓园区基础设施建设，进一步拉开框架，提高承载能力。争取全市省级工业园区新开发40平方公里以上。积极推进市开发区二次创业，重点加快城东工业园、城北新区开发；承接产业转移示范园区争取一批项目入驻；加快筹建琅琊经济开发区、定远盐化工业园两个新批省级园区；加快建设汊河工业园。突出抓好标准厂房建设。新建300万平方米标准厂房，其中市开发区百万平方米，每个县市区20万平方米以上。

　　努力提升园区档次。市开发区要争取国家级开发区、省高新区获批挂牌，天长开发区要积极创建省高新技术开发区，市承接产业转移示范园区要加快建设并成为省级一流示范园区，其他园区都要努力争取提高层次、创立省级品牌并在全省园区中实现争先进位。

　　积极申报新的省级工业园区。按照产城融合的理念，着眼城市化与工业化协调发展，科学规划园区布局，加强工业企业与生产性服务业匹配，生产与生活设施配套，把园区建设成为产业发展与生活空间协调融洽的新型城区。

　　奋力构建特色园区。大力发展配套经济、特色园区，建设各类园中园、专业园，彰显园区特色；继续推进园区合作共建，借力发展。已签约的20多个合作园区要加快建设，争取再签约一批，使共建园区发展到40个以上。加强园区招商引资，主攻重大项目，每个园区要招入1至2个20亿元以上项目。

　　切实加强园区管理。建立园区考评和奖励制度，调动各方力量，把园区建设成为产业转移的主战场、干部锻炼的主阵地、各方比试的主考场。此外，要积极开展城乡建设用地增减挂钩，继续清理闲置土地，消除违规用地，探索加强土地管理的有效办法，打好土地保障战役，满足大规模承接转移的用地需求。

　　（二）在重大项目上攻坚，推动支撑力量大增强

　　主攻重大项目，一手抓引进攻坚，一手抓落地攻坚，全力打好招商引资和项目建设两大战役。

　　突出重大项目招商，确保引资800亿。主动融入长三角，与发达地区展开点对点、线对线、面对面招商。点上招大引强，继续推进与央企和与知名民企合作，着力引进世界500强、国内500强和行业领军企业，大力引进10亿、30亿乃至50亿元以上企业。线上拉长加粗，实施产业招商、分线作战，着力培育六大支柱产业链。面上成群结队，争取抱团转移、结队落户、成群集结。同时，注重以商招商、精确招商，加强与国家级、省级和发达地区“四会”（学会、协会、商会、同乡会）、“五联”（工商联、企联、台联、青企联、侨联）联系合作，利用他们丰厚的人脉关系和同业资源，搭起更多招商桥梁；加大利用境外资金力度，主攻高技术，立足港澳台，拓展海内外；狠抓引智引研，推进产业、技术和劳动力同步转移。

　　突出重大项目建设，确保投资900亿。新开工项目3000个，争取竣工2000个（其中亿元以上100个），完成固定资产投资900亿。安排60项重点工程，实行领导联系制度，加大项目调度，狠抓“开工月”、“投产周”，实行周通报、月调度。组织千名干部帮千企。同时，继续充实项目库，对上争取，对外招商；制定“十二五”重大项目年度行动计划，确保按时间节点推进。

　　（三）在城乡统筹上推进，推动环境面貌大提升

　　把以城带乡作为统筹城乡的重头戏，打好城乡建设战役，改变环境面貌，为以工补农提供支撑，为公共服务共享创造条件。

　　奋力实现大滁城建设五大提升。主攻五个超百，全面掀起大滁城建设提升年活动，完成拆迁面积、开工建设安置房分别超过100万平方米，新建城市道路100公里，开工项目超过150个，完成投资超过150亿元。加快七大功能区建设，促进路网等公共设施大提升。城南核心区全面完成政务新区五横七纵37公里路网建设，将中都大道、南谯南路延伸至高铁站区，完成主干路网对接，使两大片区连为一体；加快龙蟠小区扩建、公租房建设，完成武警新营区、政务中心四馆等一批公共设施建设，开工建设市体育中心、市民广场、永乐路公园和市第二人民医院等，提升新区功能形象；加快CBD商务中心、发能国际等商业房地产开发，引入现代服务业，聚集新区人气。开发区、示范园区加快四纵七横路网和供电供气供热管道建设；高铁站区加快四横五纵路网建设，建成两路两站一广场，开工建设三、四水厂，实施城市综合体整体开发；科教园区打通三条主干道，开工建设城市职业学院、植物园、中央公园等；大琅琊山旅游区完成览山路建设，启动花城路观光大道和醉翁亭核心景区、东大门综合服务设施建设，加快丰乐亭核心景区、龙尾山度假中心和北大门生态停车场建设；琅琊新区加快四纵五横路网和创业服务中心、菱溪苑安置小区建设，开工建设世纪花园安置小区和菱溪夜雨湿地公园、如意湖公园。加快老城改造，促进环山抱湖沿水大提升。改造菱溪路、清流中路及周边，畅通城东、城北出口；改造南谯路和丰乐大道，提升主干道形象；打通西涧路、天长西路、紫薇南路等丁字路，改善老城路网；加快高杆灯、凤凰湖、体育场等地块改造，实施清流河两岸开发，启动南湖三期、四期改造，开工建设实验中学新校区和申花小学。加快房地产开发，促进现代服务业大提升。加快40个已审批开工的商业地产项目建设，新建续建商品房600万平方米以上。加快三个副中心建设，促进功能档次大提升。南谯新城要加快四期、五期路网和碧桂园百万平方米商业地产建设，启动新区医院、污水处理厂、双洪公园等建设；来安县要启动新区28平方公里路网建设，进一步拉开城市框架，开工建设市民广场等十大重点项目；全椒县要以滁全新区对接为目标，启动城北新区路网建设，同时打造城南新区，改造提升老城。加快推进“五城联创”，促进规划和管理水平大提升。加强规划工作，完成各专项规划编制，启动滁州市城市总体规划（2010-2030）修编，规划市老年活动中心、文化科技中心和会展中心。进一步提高规划水平。抓好城市绿化、亮化、美化、净化，建立市容环卫管理长效机制，市、区、街道、社区四级联动，分头抓好主次干道和小街巷，清除卫生死角。推进一月整治一条街活动。坚决打击违法建设。加快水电气建设。

　　强力推进县城建设“双超百”行动。天长、明光、定远、凤阳4个县城的建设项目总数要超过200个、总投资要超过150亿元。其中天长市新建续建100项城建项目，总投资39亿元，进一步拉开南市区框架，建设开发区三期、四期路网。明光市30个城建项目总投资45亿元，重点加快新城区建设，完善老城区配套。凤阳县新老城区并举，主攻新城区，实施50个以上重点城建项目，总投资30亿元以上。定远县“5个8”工程计划投资40亿元，安排32个重点项目，全面拉开城东新区8平方公里城市基本构架。6个县城都要完成规划馆建设。

　　大力实施新农村建设“千村计划”。以小城镇建设为带动，整体推进农村土地整治为契机，纵深推进新农村建设。完善镇村规划体系，加快编制村庄布点规划。以扩权强镇试点镇、人口大镇为重点，加强集镇基础设施建设，提高人口和产业集聚力。加快新农村居民点建设，扩大规模，完善配套，培育新型社区。年末新农村居民点要达到1000个。同时，积极探索降低农民进城门槛、鼓励农民创业、以土地换保障等办法，打通农民进入城镇的通道，争取新增城镇人口10万人以上，其中主城区新增5万人。

　　加快大交通为主的基础设施建设。完成交通投资18亿元。全面推进蚌淮高速凤阳段、宁洛高速来安明光间延伸段、徐明高速滁州段建设，开工建设北沿江高速公路滁马段，加快312省道天扬一级公路改建、309省道明盱段二级公路改善工程建设。继续实施农村县乡公路改造、危桥改造和农村公路安保工程，争取完成汊河集船闸改造。加快配套建设，保障京沪高铁上半年开通。继续推进滁宁轻轨项目。完成电力设施投资6.4亿元。加快“数字滁州”建设，完成通信网络投资3.5亿元。

　　（四）在工业强市上发力，推动工业经济大跨越

　　以产业规划为引导，以“三上”工程等为抓手，组织实施“12345”计划，打好工业扩张战役，争取亿元以上企业超过230家，实现50亿元以上企业的突破。

　　实施100个重点技改项目。推进天大30万吨油井管、长丰扬子4万辆皮卡及经济型SUV、康佳100万台无氟冰箱、滁州卷烟厂生产及物流基地、来安金禾化工年产15万吨硝酸、全椒欧波管业二期、凤阳玻璃日熔2200吨超厚浮法玻璃、明光润海甜叶菊、定远泉盛化工年产30万吨纯碱、天长今麦郎二期和蓝德集团新仪表生产线、南谯恒昌机械、琅琊霞客环保无染彩棉纺等100个5000万元以上的技改项目建设，确保完成技改投资200亿元。

　　力推200个重点项目投产。推动盼盼食品、申花电器、东菱电器、达世汽配、港铭钛钒合金、来安超林太阳能、全椒天和动力、凤阳确成硅化工、明光榄菊日化、天长金佩汽车零部件、南谯先进复合材料、琅琊双红新材料等200个投资3000万元以上重点项目建成投产，其中一期投资75亿元的定远华塑盐化项目要确保今年8月份投入试生产。

　　新增300家500万元以上规模企业。着力扶持一批微型企业增加投入、扩大产能，跨上500万元台阶，为2000万元以上的规模企业培育后备军。争取全年新增500万元以上企业超过300家。

　　狠抓400个重点项目开工。推动格兰仕电器、永强汽车、博西华新基地、银鹭食品、全椒旭腾光伏和君鸿纳米材料、定远中盐东兴真空制盐、凤阳福莱特超白光伏玻璃、天长天康动力锂电池和吉阳高效晶硅太阳能电池片、南谯润鑫金属、琅琊天和星光微电机等400个投资5000万元以上项目开工建设，争取南谯、天长、明光风电项目开工建设。完成全柴集团与熔盛重工重组，支持天大集团与法国瓦卢瑞克合作。

　　完成500亿工业固定资产投资。同时妥善处理好国有集体改制企业善后事宜，促进改制企业资源整合、招商引资，力求改制再生。

　　加强创新体系建设，促进转型升级。加大财政支持技术创新投入，培育引进高新技术企业。开展创新型企业试点，加强企业技术中心建设，组建和申报一批省级工程技术中心、重点实验室，争取省重大科技攻关项目。推进产学研合作，加快建设家电信息、电子器件、电线电缆、绿色照明、汽车、玻璃等检测中心和公共技术服务平台，促进科技成果转化。积极组织申报国家专利。

　　（五）在新型业态上主攻，推动服务经济大提速

　　实施服务业发展“万千百十”工程。以每年新增万户个体私营企业、早日实现千亿元服务业营业收入为目标，抓服务业年投入百亿元和每个行业十大重点项目建设，打好服务业提升战役。

　　推进十大重点市场和十大城市商业体建设。重点实施瑶海大市场、大雄农贸食品城、国际商城、奥特莱斯直销广场、红星美凯龙家居广场、滁城鲜活农产品市场、来安香江物流总部基地、定远东园大市场、南谯东雄大市场、明光万豪国际商城十大市场项目，抓好中环国际广场、城市星座、现代城、长江财富广场、盛辉99广场、南谯新碧、凤阳铸信中央城、来安新城商业综合体、全椒江海、定远大润发十大商业综合体建设。同时，发展社区商业，加强万村千乡工程建设，深入开展家电下乡与以旧换新、建材下乡等，支持邮政服务“三农”，繁荣城乡市场。

　　抓好十大景区和十大五星级标准酒店建设。加快推进琅琊山龙尾山、凤凰湖和红花湖，明光跃龙湖和栖凤湖，天长金牛湖和龙通，全椒神山，来安莲花塘，南谯大柳等十大景区建设。争取“当年小岗”竣工建成，着力打造襄河景观带。推动君家酒店改扩建升五星，提升金地大酒店五星级水平，推进凯迪豪生、喜来顿、禧徕乐、龙尾山、雅阁、红花湖、汊河碧桂园七个五星级标准酒店建设，建成全椒瑞祥、南谯碧桂园两个五星级标准酒店，同时抓好一批四星级标准酒店建设，实现四星级酒店县域全覆盖，建设一批农家乐旅游示范点；深度挖掘民俗文化，办好琅琊山庙会、全椒正月十六走太平和桃文化旅游节、凤阳花鼓旅游节等。

　　建设十大服务业集聚区和十大外贸出口基地。重点规划建设滁城老城商贸集中区、城南中央商务区（金融集中区）、信息软件园（服务外包基地）、物流园区、科教园区、大琅琊山旅游区、文化创意产业园和全椒奥康步行街、汊河综合物流园区、凤阳港区十大服务业集聚区。抓好家电、电子元器件、精细化工、玻璃器皿、机械配件、玩具、食品、纺织服装、医疗器械、特种钢管十大出口基地建设，培育博西华、天大、GLG等一批重点出口加工企业。加快海关、商检机构建设。推进出口加工区前期工作。

　　扶持培育十大重点服务业龙头企业和品牌。鼓励商之都、白云、天发广场、白鹭岛、市演艺公司等服务业龙头企业实施标准化管理，提高水平，做大做强。创新中国农民歌会运作模式，打造滁州又一响亮品牌和名片。营造同乐坊城市新客厅。培育“太守宴”、“洪武宴”、“千秋宴”等一批餐饮品牌，推出滁州餐饮文化。

　　抓好以上工作同时，大力发展金融服务业和房地产业。金融服务业，一手抓外部机构引进，争取引进1至2家股份制银行，推动徽商银行在县市设立支行。一手抓现有机构改组，加快村镇银行组建，争取2家开业；县级农村信用联社全部筹建农村商业银行；继续发展小额贷款公司、融资担保公司并扩大规模。一手抓信贷市场，创造良好金融生态环境，促进银企对接，扩大贷款规模，确保新增贷款增速超全省平均水平；推动金融服务创新，为中小企业贷款创造条件。一手抓资本市场，加强企业上市工作，争取德力玻璃、金禾化工成功上市，培育50家上市后备企业。支持保险业发展。房地产业，努力构建普通商品房与保障性住房结构合理的供应体系，争取全市房地产新开工面积超过600万平方米，开发施工面积超过1200万平方米。

　　（六）在现代农业上抓实，推动生产能力大提高

　　以转变农业发展方式为要求，大兴水利强基础，狠抓生产保供给，力促增收惠民生，着眼统筹添活力，打好现代农业战役。

　　抓好四水文章，发展民生水利。水投资，港口、自来水、水利工程、污水处理四大投资并举，力争完成18.5亿元投资任务。水安全，除险、灌溉、防洪、治污四项措施并重，开展新一轮百万亩旱涝保收农田建设提升大行动，完成3座中型水库、53座小型水库除险加固，推进骨干灌排工程建设和大型排涝泵站更新改造，加强大型灌区续建配套与节水改造，推进滁河防洪治理近期工程和高邮湖大堤加固工程建设，加大小流域、低洼地治理和清塘清渠清淤力度。水经济，积极发展滁河、淮河等港口经济和岸线经济，提升水运能力，今年重点抓好凤阳鸿运、天长祥云两个码头续建。同时，发展水上养殖和水上旅游。水保障，科学调度安排，加强城乡自来水和农村安全饮水工程建设，保证城乡生产生活用水。继续推进江淮分水岭综合治理开发。

　　抓好三大生产，推进结构调整。突出“米袋子”，加强“百亿粮仓”建设。重点开展小麦高产攻关、水稻产业提升等行动，抓好优质稻麦标准化生产，推广200万亩超级杂交稻，力争单产新提升、总产新突破。大力培育粮食品牌。突出“菜篮子”，抓好经济作物和特色农产品生产。全市建设36万亩设施蔬菜基地、12万亩甜叶菊、万亩滁菊生产基地。突出“肉案子”，主抓规模养殖，扶持千个规模养殖场，带动万个养殖户，防控重大动物疫病，促进畜牧水产养殖健康发展。推广政策性农业保险。

　　抓好四个着力，提高农业现代化水平。着力改善农业装备条件。大力发展大中型农机具和农机合作社，加强农机配套，推广机械作业；积极承接农机装备制造业转移，谋划建设农机装备制造基地。着力推动农业科技进步。完善基层农技推广体系，引进繁育推广优良品种，集成推广土地深松、精量播种、化肥深施等技术。继续开展现代农业示范区建设，争创国家级和省级示范区。着力发展现代农业产业体系。力争新引进20家以上知名农产品加工企业，新增20家以上5000万元以上的龙头企业，建设10大粮食产业园和物流园。发展图业等十大农业生态观光园，使之成为农业大观园、农村综合体、农民新舞台。林产品加工产值力争过百亿。着力推行现代农业经营方式。加强农产品质量安全工作，力争新增100个无公害、绿色和有机食品品牌。新发展200家农民合作社。增加土地流转10万亩，促进土地向种田能手集中。

　　抓好三大整治，强化农地综合开发系列效应。开展整体推进农村土地整治三年示范工作，综合整治田水路林村，争取两年20个项目近25万亩示范建设任务一年完成。同时，全面完成已报已批的6万亩建设用地置换实施任务和15万亩部省土地整理项目，促进农业生产条件改善、农村居住环境美化、农业经营方式转变。

　　（七）在改善民生上给力，推动发展成果大共享

　　坚持把保障和改善民生作为政府工作的出发点和落脚点，把富民兴滁放在更加突出位置，同步推进民生改善与社会事业发展。

　　办好惠民实事，强化民生工程。按省政府统一部署，今年民生工程调减部分项目，新增一事一议财政奖补试点、农村公路危桥加固改造、家电下乡和家电以旧换新3项，项目总数33项，总投入约25亿元。结合民生工程，重点实施“六个起来”计划。一是让群众富起来。坚持经济大发展不动摇，强化城乡居民增收基础，同时认真落实国家收入分配政策，确保城镇居民人均可支配收入和农民人均纯收入均增长12%。二是把安居建起来。实施住房保障1.4万户，新建各类保障房4280套，改造棚户区住房3715套、国有林场危旧房2919套。市本级建设安置房2万套、公租房1000套。市区完成回迁1万户。新农村居民点建成2万套。完成省下达的农村危房改造任务。三是把就业统起来。建立劳动就业与企业用工帮办组织，全面实施10万技工培训计划，争取城镇新增就业5.5万人、下岗再就业1.8万人，转移农村劳动力5.5万人。办好20个以上农民创业园。动态消除城镇零就业家庭，逐步消除农村“零转移”家庭。四是把出行便起来。改造农村公路危桥20座、渡口3个。优先发展城市公交。滁城增加52台公交车，挖掘老城区停车资源，新增800个停车位。五是把健康提起来。推行基本公共卫生服务逐步均等化，免费建立120万城乡居民健康档案，免费为20万城乡老年人提供保健服务。建设集康复、就业培训于一体的8000平方米市残疾人综合服务中心，规划建设各县市残疾人综合服务中心。改善城乡卫生环境。改造滁城27条小街巷、30座旱厕，整治100个生活大院。将农村清洁工程试点扩大到20个乡镇，对农村生活垃圾实行“村收集、乡转运、县处理”。续建16个乡镇自来水厂，修建供水管道1800公里，改善农民饮水质量。六是把保障扩起来。稳步扩大职工基本养老保险、基本医疗保险、工伤保险、失业保险、生育保险，巩固城镇居民基本医疗保险，城镇和农村居民最低生活保障做到应保尽保。抓好新农村养老保险试点。落实计划生育奖扶制度。加强市场供应与监管，稳定价格总水平，安排好群众尤其是困难群众基本生活。新建大滁城蔬菜供应保障基地2000亩。

　　加快构建与地方经济社会发展相适应的现代教育体系。实施学前教育“三年计划”，积极发展公办幼儿园，支持发展民办幼儿园。完善城乡义务教育经费保障机制，增加教育投入。推进义务教育学校尤其是农村学校标准化建设，完成101万平方米校舍安全工程。启动省职业教育改革试点市工作，着眼培养百万高素质“蓝领”工人，推进职教大市建设。大力建设滁州科教园区，加快城市职业学院、市农机校、市交通技校新校区和职业技术学院三期、技师学院建设。提高县级中职学校办学能力和水平，争创国家级示范学校。将教育费附加的30%用于职业教育。树立大职教观念，建立职业教育联席会议制度，发挥好各部门职教资源作用。支持滁州学院、安徽科技学院、滁州电大等辖区内高校建设。

　　加快建设城乡一体化的公益性医疗卫生体系。加强基本医疗保障制度建设。巩固完善新型农村合作医疗。深化基层医疗卫生机构改革，重点完善人事管理、收入分配、绩效考核等长效机制，推进乡村卫生组织一体化管理，确保药品零差价制度全面落实。加强基层医疗卫生机构队伍建设，全面完成社区和乡镇卫生院、村卫生室标准化建设任务。稳妥推进公立医院改革试点，完善以社区服务为基础的新型城市医疗服务体系。扩建市传染病医院。建设市精神病医院。抓好重大疾病防控和职业病防治。

　　加快建设公益性与市场化相结合的文化体育事业。坚持正确舆论导向，加强滁州日报和市县广播电视台建设，培育精品栏目，繁荣文化广播影视创作。加强文化基础设施建设，建设一批县文化馆、乡镇文化站，积极推广农家书屋。深入开展文化“三下乡”活动。深化文化体制改革，发展出版印刷、数字影视、演艺娱乐、文化旅游等文化产业，支持市演艺公司开拓市场。抓好街头、社区、新农村居民点等公共场所体育设施配套和市体校新校区建设。

　　（八）在群众工作上创新，推动滁州社会大和谐

　　做好群众工作是我们的优良传统和政治优势，要真正做到在感情上贴近群众，在思想上尊重群众，在行动上深入群众，在目标上造福群众，努力开展六大行动。继续开展“三民”工程“大走访”，一线掌握情况。实行“大信访”，重点推动信访工作“四个转变”，即从重来访处理转为来访与信访处理并重，首抓来信来电及时处理，防止矛盾积压、事态扩大；从重单一受访转为重综合处访，推广定远经验，全面设立县市区“信访大厅”，组织矛盾纠纷和信访问题较多的单位进驻；从重包案转为重息案，下决心解决一批重复访和信访积案难案；从重信访处理转为重信访监督，督促广大公务员强化政策观念，公开公正办事，把矛盾化解在日常工作中。探索“大巡防”，加强警务工作信息化建设，推进城市监控设施、城市周边治安卡口、治安警亭联网化，“110”、派出所、社区治安点一体化，警力下沉，充实一线，推进平安滁州建设。实施“大挂牌”，全面排查道路、矿山、企业、校园等领域和消防工作存在的安全隐患，公开挂牌整治，坚决防止和遏制重特大事故发生。试点“大计生”，深化“村为主”，推进人口计生基层群众自治，改进农村流动人口计生管理，严控计划外生育和人口性别比，稳定低生育水平。组建“大平台”，建设政府系统数字应急平台，提高突发事件一线联动反应能力。同时，加强地质、旱涝等自然灾害应对和防灾救灾、防震减灾工作。加强市场信用体系建设，维护市场秩序。加强农产品质量检测，强化食品药品监管。启动新一轮普法，认真做好法律援助工作。完成第八届村委换届选举，深化村务公开。扎实做好优抚安置和“双拥”工作，争创双拥模范城全国“四连冠”、全省“七连冠”。完成第六次人口普查、第二轮地方志编修和档案馆标准化建设。认真做好国防动员、人民防空、民族宗教、外事、侨务、对台、保密、关心下一代等工作，支持工会、共青团、妇联、工商联、科协、红十字会工作，发展妇女儿童、老龄人、残疾人等各项事业。

　　（九）在持续发展上尽责，推动生态环境大改善

　　开展节能技术改造，继续淘汰落后产能。抓好公共机构节能。加强城市污水管网建设，严格在线监控设施管理，提高污水处理厂运行水平。加强减排重点领域、重点项目、重点企业专项治理。整治农业源污染和汽车尾气污染。认真执行环境保护“三同时”制度。继续创建国家环保模范城。推进生态市建设，强化资源保护。完成植树造林15万亩。加快国有林场改革和林权制度改革，开展森林采伐管理改革试点。推进采矿权出让审批制度改革，整顿开发秩序，促进资源整合，提高利用水平。引导企业开展清洁生产和废弃物综合利用，发展循环经济。依法加强基本农田保护、土地复垦开发和土地管理，确保建设占用耕地占补平衡。编制全市水源地保护规划，加强重点水源地保护。

　　四、努力建设奋发有为、人民满意的服务型政府

　　适应社会转型的新形势、加快发展的新要求、人民群众的新期待，不断提高政府工作水平。

　　进一步完善依法规范行政的体制。自觉接受市人大及其常委会的法律监督和工作监督，主动接受市政协和各民主党派、工商联与无党派人士民主监督，虚心接受人民群众和舆论媒体的社会监督，认真办理落实人大议案、代表建议和政协提案，充分发挥工会、共青团、妇联、科协、残联等人民团体作用。以建设法治政府为目标，全面贯彻落实国务院依法行政实施纲要和加强法治政府建设的意见，加强制度建设，规范权力运行。完善行政复议和相对集中行政处罚制度，继续推进政务公开，重点开展执法流程网上办理、建立自由裁量权基准制度、执法评议公开考核三个创新，规范执法行为，促进公开公正、透明文明执法。

　　进一步健全民主科学决策的机制。健全专家论证、合法性审查、社会公示和听证制度，重点探索公众参与政府规范性文件制定，政府规范性文件实施情况社会评估，部门论证、专家咨询、公众参与、专业机构测评相结合的决策风险评估等机制，使各项决策更加贴近民意、适应形势、减少失误。探索推进网络问政，办好"12345"市长热线、政风行风热线、滁州民声网、市长信箱，拓宽沟通民意渠道。

　　进一步提升推动科学发展的能力。着力培育乐于奉献、勇于担当、善于作战的公务员队伍，重点抓好三个改进。改进知识结构，提升创新发展能力。今年起每年组织千名公务员通用知识和专业知识大培训，分类展开，加强考评。改进绩效管理，提升推动工作能力。开展百项指标定期大考评，促使广大公务员时刻感受到压力，进一步强化动力，钻进去深入调研，跳出去研究周边，跳起来多“摘桃子”。改进思维方式，提升风险掌控能力。善待社会的知情权和表达权，正确对待舆论批评，把握正确舆论导向，研究有效防范和化解社会热点问题的办法途径。

　　进一步强化为民施政的意识。深入开展“服务项目、服务企业、服务创业、服务民生”四项活动。服务项目，继续推进行政审批制度“两集中、两到位”改革，精简审批流程，压缩审批时间，推进管批分离，规范审批行为，扩大跨部门联合审批覆盖面，把更多审批项目纳入行政服务中心，引导规范中介服务。服务企业，真正把企业看作创富主体，把企业家当作社会宝贵财富，深入开展企业帮扶帮办活动，多服务，治刁难。服务创业，组织实施好全民创业战役，进一步清理束缚全民创业的规章制度，营造创业光荣的良好氛围，降低门槛，加强扶持，搭建平台，培育更多创业主体。服务民生，要强化群众观念，认真执行政策，及时解决群众反映的困难和问题。进一步加强效能督查、明查暗访、行政问责，促进作风改善、形象提升。

　　进一步树立勤政廉政工作的风气。严管重点领域和关键岗位，重点抓好公共资源交易和招投标管理，继续治理工程建设领域存在的突出问题。严管政风行风，重点治理教育收费、公路“三乱”、中介服务收费、“四项资金”等，纠正违规审批，清理违规收费，继续开展行风评议活动。严管财经支出，加强国有资产管理，规范“收支两条线”管理，压缩一般性支出，制止铺张浪费，建设节约型机关。严管公务员队伍，突出抓好政府系统惩治和预防腐败体系建设，加强政府内部的审计和行政监察监督，主动接受外部各方监督，严肃查处违纪违法案件。

　　各位代表，“十二五”美好画卷正在展开，进军号角已经吹响！我们的使命光荣而艰巨。让我们紧密团结在以胡锦涛同志为总书记的党中央周围，高举中国特色社会主义伟大旗帜，全面贯彻落实科学发展观，在省委省政府和市委的坚强领导下，振奋精神，坚定信心，凝心聚力，扎实工作，为滁州更加美好的明天而努力奋斗！
